# The Thorn in the Dyad: A Vision on Parent-Child Relationship in Autism Spectrum Disorder

**DOI:** 10.5964/ejop.v14i3.1453

**Published:** 2018-08-31

**Authors:** Teresa Del Bianco, Yagmur Ozturk, Ilaria Basadonne, Noemi Mazzoni, Paola Venuti

**Affiliations:** a Department of Psychology and Cognitive Science, University of Trento, Rovereto, Italy; b Department of Brain and Behavioral Science, University of Pavia, Pavia, Italy; Department of Psychology, Webster University Geneva, Geneva, Switzerland; University of South Wales, Newport, United Kingdom

**Keywords:** Autism Spectrum Disorder, parent-child relationship, non-verbal communication, parental stress, intervention

## Abstract

Parents and children form a family: their characteristics balance personal and family well-being with healthy levels of stress. Research on parents of children with Autism Spectrum Disorder (ASD) demonstrated that higher levels of parental stress are associated with communication impairment, a core symptom of ASD. The aim of this article is to discuss the connection between non-verbal communication impairment and parental psychological distress, in families with children with ASD. The interaction between atypical communication and distress of parents likely determines a cascade effect on the parent-child dyad; in fact, it decreases the quality and frequency of interactions, preventing the establishment of a healthy parent-child relationship and leading to a series of collateral problems. To this perspective, guiding the parents to reframe their children’s atypical communicative behaviour can relieve parental stress and re-program the interactional routine. This observation stresses the importance of interventions centred on the dyad, especially during early development and soon after the diagnosis, when the communicative impairment may be extremely severe.

Autism Spectrum Disorder (ASD) is a neurodevelopmental disorder characterized by impairments in social communication and restricted, repetitive patterns of behaviour and interests (American Psychiatric Association, 2013). An insufficient predisposition of the child to engage in communication with her parents significantly affect the parent-child relationship. From the earlier stages of development, non-verbal impairments of communication are present in children with ASD (Mundy, Sigman, Ungerer, & Sherman, 1986) and contribute to lessening interactional encounters. Research has been carried out on communication difficulties, but researchers have not treated the connection between difficulties in parenting children with ASD and non-verbal impairments. Our aim is to illustrate the connection between non-verbal communication channels (i.e. cry, social gaze, gestures and movement), and a problematic parent-child relationship.

For selecting the papers relevant to our overview, we undertook a bibliographic research on online databases (i.e., Web of Science, Scopus, Discovery). We based our search on keywords pertaining to the topics: pre-verbal communication (e.g., “cry”, “gaze-following”, “gestures”, “biological motion” and “autism spectrum disorder”), parental stress (e.g., “parental psychological distress”, “parenting distress” and “autism spectrum disorder”), gastrointestinal disturbances (e.g., “gastrointestinal symptoms”, “food selectivity”, and “autism spectrum disorder ”) and interventions grounded in the parent-child interaction (e.g., “parent-child intervention”, “autism spectrum disorder”). The research comprised a number of suitable synonymies of the search terms. We included in our revision quantitative peer-reviewed research articles. As selection criteria, we considered their relevance to the aforementioned topics and the year of publication (we included articles published from the year 2000, with the exception of 8 historical references).

The first section offers an overview of the impairment of the non-verbal communication channels in ASD from the early stages of development. Afterwards, the character of difficulty of the parent-child dyad will be introduced with an example of a problematic situation, the delayed diagnosis of gastrointestinal disorders and its inefficient management (Buie et al., 2010). In the light of the evidence showing the importance of a good quality of communication between parents and child, a strong intervention on parents’ and children’s communication abilities is justified. Finally, we will describe models of intervention for parents of children with ASD, based on empowering the parents’ ability to read their child’s communicative attempts.

## Preverbal Channels Impairment: Cry, Gaze, Gestures and Movement

It is a truth universally acknowledged that a new-born will cry when distressed (Zeskind & Lester, 2001) and look at your face (Johnson, Dziurawiec, Ellis, & Morton, 1991) from the very first moments of his/her life. Cry delivers messages about needs to the caregiver (Zeskind & Lester, 2001) while gazing into others’ face and eyes helps the infant engaging with others (MacPherson & Moore, 2007) and regulating emotions and arousal (Hsu, Fogel, & Messinger, 2001). Infants’ also preferentially attend to biological movement, typical of persons (Simion, Regolin, & Bulf, 2008). Before the first birthday, the infant even produces conventional gestures (Messinger & Fogel, 1998).

Children with ASD express atypical patterns of distress vocalization, such as higher fundamental frequency (f0), shorter inter-bout pauses, and fewer utterances (Esposito, Nakazawa, Venuti, & Bornstein, 2013; Esposito, Venuti, & Bornstein, 2011; Sheinkopf, Iverson, Rinaldi, & Lester, 2012). Retrospective analysis often detects a lack of eye-contact before the age of diagnosis (Baranek, 1999). With the modern eye-tracking technique, research unveiled that children with ASD visually avoid faces that display direct gaze (i.e., gaze directed forward to the observer; Senju & Johnson, 2009). The importance of the integration between non-verbal signals is evident in at-risk children, the siblings of children diagnosed with ASD, that have an increased risk of developing the condition themselves because of shared genetic background (the estimated risk raises to ~7% in large population studies, versus 1% in unrelated individuals; Baird et al., 2006; Grønborg, Schendel, & Parner, 2013). In at-risk infants, scarce eye-contact and reduced gestures have been enlisted as markers at 18 months with a high predictive power for future diagnosis (Chawarska et al., 2014). In particular, the absence of eye contact, sharing behaviours and descriptive gestures during social exchanges corresponded to a triplicated risk of diagnosis. Additionally, this study highlighted the relevance of the interactive context, such as play, whose impaired establishment is part of the defining profiles (Chawarska et al., 2014).

With regard to the ultimate non-verbal communication channel – movement – studies have revealed that children with ASD show a dual deficit, both on the productive and the receptive side of body-language. In fact, toddlers and children with ASD rarely express their intentions and feelings through gestures and body movements (Dziuk et al., 2007; Trevarthen & Delafield-Butt, 2013;). Furthermore, a number of studies report that the general motor skills are impaired, such as gait, sitting and fine grasping (Esposito, Venuti, Apicella, & Muratori, 2011; Esposito, Venuti, Maestro, & Muratori, 2009; Lloyd, MacDonald, & Lord, 2013). On the receptive side, data indicate that children with ASD fail to orient towards others’ movement (Annaz, Campbell, Coleman, Milne, & Swettenham, 2012; Falck-Ytter, Rehnberg, & Bölte, 2013; Klin, Lin, Gorrindo, Ramsay, & Jones, 2009).

Children with ASD show less conventional-interactive gestures, compared to children with Down Syndrome and typical development (Mastrogiuseppe, Capirci, Cuva, & Venuti, 2015), and respond less to communicative gestures, such as lateral gaze and pointing (Falck-Ytter, Fernell, Hedvall, von Hofsten, & Gillberg, 2012). Cognitive functioning, as well as the severity of the symptoms, are specifically associated with the expression through gestures, rendering this behaviour a relevant marker of the evolution and outcome of the condition (Mastrogiuseppe, Capirci, Cuva, & Venuti, 2015). Besides, patients with ASD scarcely integrate different kinds of expressive behaviours, such as gaze and gestures, and barely coordinate with their social partner (Rozga et al., 2011). In this particular study, the authors reported significant differences in responding to pointing gestures, pointing and handling objects to initiate an interaction already at 12 months. Further details on early, non-verbal communicative impairment in ASD are provided in Table 1.

**Table 1:** Overview of Non-Verbal Cues, Adaptive Functions and Impairment in ASD

Nonverbal cue	Typical Emergence	Function	Impairment in ASD
Cry	From birth	Cry carries information that affects the infant-caregiver relationship; adults react differently to the cries of high-risk or low-risk babies (LaGasse, Neal, & Lester, 2005; Zeskind & Lester, 1978)	Atypical patterns of distress vocalization: higher fundamental frequency, shorter inter-bout pauses, fewer utterances (Esposito, Nakazawa, Venuti, & Bornstein, 2012; Esposito et al., 2013; Oller et al., 2010)
Eye-Contact (EC)	Days from birth: preference for eyes with a direct gaze (Farroni, Csibra, Simion, & Johnson, 2002)	At 6 months, EC facilitates gaze-following (Senju & Csibra, 2008). At 9 months, EC plays as a hint for triadic interactions (Joint Attention) (Rochat, 2014)	Drop of visual attention allocated to the eye-region between 2 and 6 months in at risk infants, that later receive the diagnosis (Jones & Klin, 2013)
Gaze-Following (GF)	2-4 months: emerging GF; 6-8 months: stabilization (Gredebäck, Fikke, & Melinder, 2010).	GF scores at 10.5 months predict the use of mental-state words at 2.5. years At 12 months, GF establishes affective referencing to objects (Itier & Batty, 2009) GF skills at 12 months predict later productive vocabulary (Tenenbaum et al., 2015)	Less allocation of attention to gazed-at-objects in experimental settings (Bedford et al., 2012; Falck-Ytter et al., 2012; Freeth, Chapman, Ropar, & Mitchell, 2010)
Gestures	4 months: non-directed pre-reaching (Blake, O’Rourke, & Borzellino, 1994) 12 months: effective pointing (Blake et al., 1994)	At 9 months, infants focus on objects out of field of view, after the parent looks and points at it (Flom, Deák, Phill, & Pick, 2004) Gestures prefigure referential labeling, before language is developed (Liszkowski, 2008)	At 24 months, gestures production differs in terms of frequency and quality: less conventional-interactive, pointing and nominal gestures; more ritualized request gestures (Mastrogiuseppe et al., 2015)
Biological Movement (BM)	After birth: detection of Point Light Display representing BM (Bardi, Regolin, & Simion, 2011)	The perception of BM is correlated with action understanding and intention inference (Blakemore & Decety, 2001)	Atypical kinematic (Cook, Blakemore, & Press, 2013) Difficulties in chaining motor acts (Cattaneo et al., 2007) and recognizing emotions (Nackaerts et al., 2012)

The atypical emergence and development of non-verbal communicative abilities may prevent parents from being aware of the child’s needs. In the next section, we will highlight the relationships between parental stress and characteristics of ASD and the specificity of preverbal communication impairment as a child-related stressful factor.

## Raising a Child With ASD: Parents’ Psychological Distress and Communication Difficulties in ASD

Parents of children with ASD are the first people to recognize the difficulties and differences in their children and are responsible for their education and treatments. Hence, besides the study of individuals with ASD, the impact of the difficulties in communication on the well-being of parents is of greatest importance. The challenge of being parents of children with ASD has been addressed by several studies that report that parents of children with ASD experience elevated levels of stress related to parenting a child with ASD (Ingersoll & Hambrick, 2011; Ozturk, Riccadonna, & Venuti, 2014). Compared to parents of typically developing children and with others developmental disabilities, parents of children with ASD report more psychological distress and mental issues, such as depression (Eisenhower, Baker, & Blacher, 2005; Estes, Munson, Dawson, & Koehler, 2009; Ingersoll & Hambrick, 2011; McStay, Trembath, & Dissanayake, 2014). In one study, a striking percentage of parental distress (85%) resulted related to the parent’s role (Ingersoll & Hambrick, 2011). The characteristics of a child and a family are associated with parenting distress (Bishop, Richler, Cain, & Lord, 2007; Hastings et al., 2005; Lecavalier, Leone, & Wiltz, 2006). The child’s ability in communication has a determining impact on parents’ well-being (Bebko, Konstantareas, & Springer, 1987; Ekas & Whitman, 2010) and parents of children with ASD (Lecavalier et al., 2006) and intellectual disability (Hassall, Rose, & McDonald, 2005) with better communication skills report lower levels of stress. In fact, parents’ behaviour can be significantly modified by the child’s condition: difficulties in communication makes the parents anxious and worried (Marcus, Kunce, & Schopler, 2005) and unusual language and communication pose difficulties for parents when they spend time with their children (Estes et al., 2009), resulting in psychological distress. On the other hand, better communication in the everyday context has a positive impact on the parenting sense of competence (Ozturk et al., 2014). The extent of the stress connected to communication impairment has been tested by investigating the effect of treatment-related changes in a child’s communication ability on parents’ well-being (Ozturk, Vivanti, Uljarevic, & Dissanayake, 2016): mothers reported higher satisfaction, mediating the level of psychological distress, when their children’s communication ability improved.

### Parent-Child Relationship

Conspicuous evidence connect perceived parental stress and maladaptive parenting strategies with specific characteristics of ASD. Factors of the Parental Stress Index (PSI; Abidin, 1995) has been found to correlate with child’s problematic characteristics that relate to the communicative domain: scores estimating general and parenting-related distress strongly correlate with the Social Responsiveness Scale (Zaidman-Zait et al., 2011) and the Communication Scale of the ADOS-G (Ozturk et al., 2014). The perception of issues related to the child, estimated by the subscale Difficult Child, correlates with scores on the Vineland Adaptive Behaviour Scale, including Communication (Hassall et al., 2005) and communication as a problematic behaviour predicts PSI variance over one year of observation (Lecavalier et al., 2006). Communication impairment affects perceived parenting difficulty and the levels of stress of parents. Furthermore, communicative competence has the long-term predictive power of parents’ well-being. Keen and colleagues (2010) reported that scores of the Communication and Symbolic Behaviour Scales – Developmental Profile (CSBS-DP), a caregiver questionnaire about parent’s perception of child’s communicative behaviour, showed a significant amelioration when the family was supported by a short-term, intensive intervention mediated by a trained professional. This intervention resulted efficacious in reducing child-related parental stress (Keen et al., 2010), but the follow-up, standard assessment did not detect a significant degree of improvement. Thus, it is possible that the intervention promoted the reframing of the child’s behaviour from the parents’ point of view. Another study (Baker-Ericzn, Brookman-Frazee, & Stahmer, 2005) examined changes in PSI of parents with a toddler with ASD before and after an intervention inclusion program: they found that the Child Domain improved for mothers (but not in fathers). Indeed, it has been found that mothers are positively affected by their child’s communication skills in everyday life contexts (Ozturk et al., 2016). From this brief overlook, it is clear that communication influences the development of an effective relationship between parents and their daughter or son. In the case of ASD, understanding the child’s requests is difficult for parents that become confused observers of his or her discomfort. A sense of helplessness may come along and largely frustrate parents’ attempt. Non-verbal communication is the primary way of expression of distress and has a pivotal importance in young toddlers, non-verbal children and their parents. In the next paragraph, we are going to describe a situation where the non-verbal expression of distress confound parents and physicians.

### Consequences of Communication Impairments: Management of Gastrointestinal Problems and Food Selectivity

Gastrointestinal problems are common in children with ASD but difficult to detect, given the deficit in communication that complicates the description of symptoms and/or the expression of pain even through intentional non-verbal communication (Buie et al., 2010). In fact, behaviour that parents usually report as “unexplained” can actually express gastrointestinal discomfort: vocalizations (i.e. frequent clearing of the throat, screaming, groaning) and motor behaviour, such as facial grimacing, application of pressure to abdomen, unusual posturing, and an increase of repetitive behaviour (Carr & Owen-Deschryver, 2007; McAtee, Carr, Schulte, & Dunlap, 2004). Moreover, it is possible that children with compromised communication skills perceive higher levels of stress and this could affect the gastrointestinal tract. In this context, finding the way to overcome the difficulties in the diagnosis of gastrointestinal disorders among ASD children is clearly of major importance, especially for those who have strong communication impairments. This advancement would possibly have a beneficial effect on parental stress, reducing their sense of powerlessness.

Another problematic behaviour possibly related to communication impairments is food selectivity, that is the limited food repertoire and high-frequency single food intake (Bandini et al., 2010). Several studies indicate a considerable incidence of food selectivity among autistic children (Ahearn, Castine, Nault, & Green, 2001; Bandini et al., 2010; Schreck, Williams, & Smith, 2004; Williams, Gibbons, & Schreck, 2005). Food selectivity is part of the everyday management of feeding problems in ASD and challenges the caregivers (Dominick, Davis, Lainhart, Tager-Flusberg, & Folstein, 2007), since problematic mealtimes and a negative impact on dietary habits of other members of the family have been reported (Curtin et al., 2015). Parents of children with ASD and food selectivity tend to show higher levels of parenting stress (Postorino et al., 2015) and even spousal stress (Curtin et al., 2015). Problems in communication might contribute to this situation. For example, parents might unintentionally force the child to eat something that he/she has problems to digest properly and they cannot intervene promptly to prevent the extension of selectivity to other foods of the same category. In conclusion, a defective non-verbal communication, exemplified by discomfort and symptom expression, can heavily contribute to frustrate the parents’ attempts to manage the everyday life with their child with ASD.

## What to do? Models of Intervention for Parents and Children With ASD

For a successful management of a child with ASD, intervention should support parenthood and deal with the psychological distress of parents. As we previously explained, the child’s difficult predisposition to communicate significantly influence the relationship with the parents, that in turn feel high levels of stress connected to the child and their role. Parents should be trained by professionals to use their knowledge about their special child in various situations, such as during lunchtime or in the playground. Empowered parenting skills can significantly improve caregiver-child interactions, which is the basis of the several situations parents experience with their child diagnosed with ASD.

The interaction between a child and his/her parents is important and crucial in the daily life of the family. However, atypical child development significantly alters the modalities of interaction between parent and child (Venuti, Bentenuto, & Villotti, 2011). In a broader sense, activities of children with ASD can stress parents’ attempts to frame and build interactive behaviour (Mundy, Sullivan, & Mastergeorge, 2009). The question arises about how atypical development has such specific consequences on parenting strategies. Coming back to the first communication media, the properties of cry have been linked to the quality of the first interactions: for instance, atypical cry episodes prevent the caregiver from efficaciously responding to the infant’s distress (Venuti, 2003; Venuti & Esposito, 2007). The pace is short towards feelings of inadequateness and disruption of parenting practices. Qualitative differences have been observed in the interactive style of parents with their children with ASD, compared with parents of typical children and other disabilities, such as Down Syndrome (Venuti et al., 2011). Namely, parents of children with ASD display more behavioural attempts of control, physical contact, and similarity between mother’s and father’s parenting style (Venuti et al., 2008; Venuti et al., 2011). It is worth noticing that these parents often show sufficient levels of “intuitive parenting” (Papoušek & Papoušek, 2002), though their behaviour becomes maladaptive due to the modified interaction they are exposed to. The good quality of a caregiver-child relationship can be measured, for instance, considering the levels of synchronization. The level of synchrony during natural, unstructured social exchanges, like joint play, is a measure of one’s own behavioural and mental plasticity in front of a social partner. The ability to understand others’ attitude and react contingently makes the interaction synchronized, accessible and fluid. If the child with ASD needs support in understanding his or her partner in this context, so does the parent. A high skilled parent could render the interaction less effortful and facilitate the child’s development in several domains. In fact, a more efficient parental style, with normative levels of synchronization, has a positive impact on the developing child, influencing, for instance, language development (Siller & Sigman, 2002; Siller & Sigman, 2008). Therefore, parents’ adequate sensitiveness has to be considered an incontrovertible aspect of a successful therapeutic approach. The same principle is valid for many situations of everyday life that take place out of the clinic: lunch and dinner, eating meals, with the problems that we described before. If the parents are able to read the signs of their child, they will be ready to interpret the source of distress and make correct choices. It is reasonable to think that parents should be educated to use their knowledge about their special child in various situations, and not only in predefined therapeutic settings. Therefore, the support for parents of a trained professional should be, if not continuous, constant in time and set on the new challenges along the child’s development.

The therapeutic approach should not impose specific response to parents but offer a safe standpoint for reframing the child’s behaviour (Bornstein & Venuti, 2013; Fava et al., 2011; Venuti, 2012). A reasonable option is that parents observe the child’s communicative modalities during an interaction with a therapist. From this neutral position, a parent can observe what behaviour conducts to failure, intuitively guess and develop a knowledgeable perspective. This process is natural and devoid of negative consequences on the emotive sphere of the parent, avoiding additional disruption of his or her self-efficacy. The mediation of parent-child interaction by the therapist is the next step. During this phase, difficulties and worries can be verbalized and new approaches to a resolution discovered. Direct observation is only one aspect of a complex and complete series of interventions: through colloquia, deferred observation of recordings of therapeutic sessions, and the participation to groups for sharing experiences, a satisfying level of parenting competence can be achieved. Clearly, one of the goals of this kind of intervention is to support parenthood. As it has been recently shown, better communications skills of the child significantly affect parents’ psychological distress (Ozturk et al., 2016); moreover, parents’ expertise in the child’s special way of communicating possibly improves after a number of cycles of treatment.

### Future Directions

We took on this theme and highlighted some connection with the clinical picture of ASD; however, in order to really face this topic, it is necessary to take into account also differences among parents. For instance, gender specificity of parental reactions should be evaluated: the majority of studies included mothers, but not fathers. Recent findings showed that levels of general stress related to parenting a child with ASD are not different between fathers and mothers (Ozturk et al., 2014). However, a specific scale of the Autism Diagnostic Observation Schedule-Generic (ADOS-G), the Communication scale, resulted positively correlated with fathers’ perceived stress related to the child and with mothers’ scores of general stress (Ozturk et al., 2014). Therefore, an open question is if distress related to non-verbal communication impairment in ASD differs between mothers and fathers since a difference in terms of parental attitude is present. The scores associated with social exchanges have been reported higher in mothers of children with ASD (Ozturk et al., 2014); social exchanges involve face-to-face interaction and a closer physical contact, factors that could modulate differently the impact of an impaired non-verbal communication. This could be a starting point for conceiving future research projects and differentiated intervention programs.

## Conclusion

In this paper, we illustrated the importance of nonverbal communication impairment –including cry, social gaze, and movement execution and comprehension – in modulating the parent-child relation. Through a relevant issue for families of children with ASD – the detection and management of gastrointestinal problems and food selectivity – we described an example of the negative consequences of non-verbal communicative impairment in the everyday life. In addition, we discussed how parental distress relates to different degrees of functioning in communication between parent and child. Finally, we presented an intervention strategy that may help parents to overcome the communication difficulties.

To conclude, the intervention with the child on early communication channels is crucial soon after the diagnosis, and parents should be instructed about the atypical communication attempts. A better communicative attitude in the child and an empowered sensitivity of parents to atypical attempts of communication has the potential to improve the quality of the parent-child relationship, reducing parental stress and ameliorating the life of families. Further research is needed in order to find out the best way to work on non-verbal communication aspects as well as how to adapt these kinds of interventions to the child and parents’ needs.
